# Simulation of Inference Accuracy Using Realistic RRAM Devices

**DOI:** 10.3389/fnins.2019.00593

**Published:** 2019-06-12

**Authors:** Adnan Mehonic, Dovydas Joksas, Wing H. Ng, Mark Buckwell, Anthony J. Kenyon

**Affiliations:** Department of Electronic and Electrical Engineering, University College London, London, United Kingdom

**Keywords:** neuromorphic, RRAM, inference, silicon oxide, machine learning

## Abstract

Resistive Random Access Memory (RRAM) is a promising technology for power efficient hardware in applications of artificial intelligence (AI) and machine learning (ML) implemented in non-von Neumann architectures. However, there is an unanswered question if the device non-idealities preclude the use of RRAM devices in this potentially disruptive technology. Here we investigate the question for the case of inference. Using experimental results from silicon oxide (SiO_*x*_) RRAM devices, that we use as proxies for physical weights, we demonstrate that acceptable accuracies in classification of handwritten digits (MNIST data set) can be achieved using non-ideal devices. We find that, for this test, the ratio of the high- and low-resistance device states is a crucial determinant of classification accuracy, with ~96.8% accuracy achievable for ratios >3, compared to ~97.3% accuracy achieved with ideal weights. Further, we investigate the effects of a finite number of discrete resistance states, sub-100% device yield, devices stuck at one of the resistance states, current/voltage non-linearities, programming non-linearities and device-to-device variability. Detailed analysis of the effects of the non-idealities will better inform the need for the optimization of particular device properties.

## 1. Introduction

Computation is at a crossroads thanks to the exponential growth in often complex and noisy unstructured data (data not in easily-accessible numerical form, but unorganized and text- or image-heavy), and the ever-increasing demand for systems to process it. As examples, the Internet of Things (IoT), Big Data, autonomous vehicles, and Artificial Intelligence (AI) pose severe challenges to the speed and power consumption of existing computing systems and suggest that we should change our approach. Present day von Neumann computing architectures require constant shuffling of data between memory and processing units, providing a critical performance bottleneck (McKee, 2004). Most clock cycles are wasted in moving data rather than computing, while physical separation of memory and processing builds in latency. In recent years, brain-inspired computing, as a potential solution to this pressing challenge, has gained significant attention. The main paradigm shift is in breaking the physical separation between memory and processing using novel non-von Neumann architectures (Wright et al., 2013). Closely related is a neuromorphic approach (Mead, 1989, 1990; Poon and Zhou, 2011), that draws inspiration from the human brain, which is a remarkable power-efficient system—many orders of magnitude better than the current most power-efficient CMOS systems.

Artificial neural networks (ANNs) are computing systems that take some inspiration from biological neural networks. ANNs consist of neurons and adjustable connections between them—synapses—that store all the information about the network. There are two phases during the operation of ANNs: training and inference. During training, learning algorithm adjusts the synaptic weights of an ANN by exposing it to large data sets. Such a trained network is capable of inferring previously unseen data and produce the desired outcome during the inference phase.

Although applicable to many problems, large ANNs can be time- and power-consuming when implemented on general purpose von Neumann computers that are fundamentally digital, thus it is advantageous to consider analog alternatives. The most crucial part of realizing ANNs in hardware (i.e., in physical neural networks (PNNs), rather than in software) is the implementation of synapses (physical weights) as they are responsible for most of the computations in an ANN. Realization of synapses using standard CMOS components is impractical; continuous synaptic weight adjustment is preferred, which makes binary components intrinsically unsuitable. Thus, the most important component in most neuromorphic systems is an analog memory device that exhibits multiple state programmability, high power-efficiency and high density. Many such devices have been considered in recent years, including phase-change memories (PCM) (Kuzum et al., 2011; Nandakumar et al., 2018; Sebastian et al., 2018) and resistive random-access memories (RRAM) (Chang et al., 2011; Yu et al., 2013b; Serb et al., 2016; Stathopoulos et al., 2017; Pi et al., 2019), both of which may be considered to be subclasses of memristive systems.

Resistive RAM technology is based on simple two terminal (metal-oxide-metal) nanodevices whose resistance can be repeatedly varied, with low operational energy and very high levels of integration (Torrezan et al., 2011; Mehonic et al., 2012). One of the most important properties of RRAMs is that they exhibit synapse-like plasticity. As a result, different learning laws have been realized using these devices, e.g., spike-timing-dependent plasticity for spiking neural networks (Jo et al., 2010; Serrano-Gotarredona et al., 2013; Yu et al., 2013a; Chang et al., 2016). Although neuronal activation is typically realized in CMOS technology while RRAMs are used solely as models of synapses, there have been demonstrations of memristive devices exhibiting neuronal functionalities (Pickett et al., 2013; Mehonic and Kenyon, 2016; Stoliar et al., 2017), resulting in further power consumption reduction. Some impressive proof of concept work has been conducted, achieving supervised (Prezioso et al., 2015) or unsupervised learning (Serb et al., 2016) using RRAM-based crossbars as PNNs (Ielmini, 2018).

Furthermore, RRAM crossbars intrinsically represent physical matrices and have an innate capability to compute approximate matrix-vector product, a key mathematical operation in many machine learning (ML) algorithms, in a constant time step. These approaches, although still at an infant stage, provide a promising route in achieving speed and power efficiency improvements of many orders of magnitude compared to today's state-of-the-art microprocessors (Gokmen and Vlasov, 2016).

Nevertheless, the imperfection both at the device (variability, a limited number of multiple resistance states, a small operational range of resistance modulation, non-linearity of voltage-current characteristics, non-linearity of resistance modulation with voltage pulses) and system levels (sneak currents and high resistances of interconnections in crossbar arrays) cannot be ignored and can lead to reduction in computational accuracy (Burr et al., 2015; Chai et al., 2018).

In this paper, we simulate how the various non-idealities of RRAM devices affect the inference accuracy of trained neural networks when they are physically implemented by utilizing RRAM-crossbar arrays. First, we present and discuss experimental results obtained from SiO_*x*_-based RRAM devices in the context of their use as proxies for weights in ANNs implemented on crossbars. Then we analyse the effects of device non-idealities on inference accuracy by simulating those non-idealities on ANNs that were firstly trained on the MNIST handwritten digits data set (LeCun et al., 2010) using conventional software methods. It is critical to understand the effects of the devices' non-idealities in order to better inform the optimization of specific device properties.

## 2. Materials and Methods

### 2.1. RRAM Experiments

We use our SiO_*x*_ RRAM devices as a case study to quantify the non-idealities that are common to all RRAM devices. Our devices consisted of 35 nm thick sputtered SiO_*x*_ layers (x≈1.9) sandwiched between molybdenum bottom electrodes and gold top electrodes, with a 3 nm thin titanium wetting layer to ensure adhesion of the gold to the oxide. We investigated the devices using two main approaches: I/V sweeps and application of voltage pulses. I/V sweeps are useful for quantifying the amount of I/V non-linearities, while applying voltage pulses helps us understand the extent to which it is possible to change the resistance of RRAM devices in a controlled and continuous manner, as well as achieve high dynamic ranges, i.e., high ratios between the highest and lowest resistances.

### 2.2. Simulations

In our analysis we train ANNs using conventional software methods and then simulate the effect on inference accuracy when the synaptic weights are mapped onto RRAM devices with non-ideal characteristics, some of which have been extracted from the experiments described in subsection 2.1. We do not consider *in-situ* learning in this analysis.

**Figure 1:**
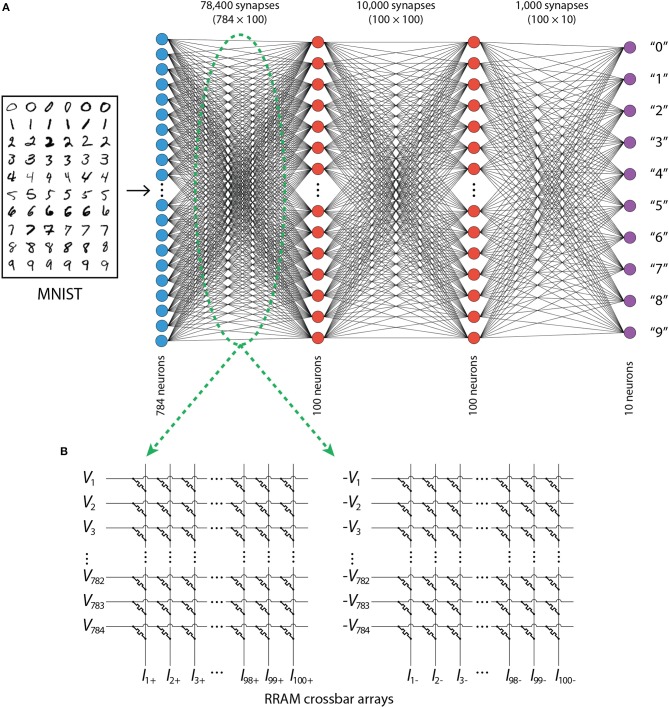
**(A)** Architecture of ANNs. **(B)** RRAM-based crossbar arrays used for the implementation of weights in the ANN.

ANNs with fully connected neuronal layers were trained to recognize handwritten digits (using the MNIST data) by utilizing backpropagation algorithm. For convenience, and given that multiple networks had to be trained for averaging purposes, only the simplest training approaches were used, therefore the accuracy of ANNs with continuous weights is not state-of-the-art, but rather in the range of 97–97.5% for most of the architectures explored. Our focus is not to achieve state-of-the-art accuracy, but to understand how inference accuracy *changes* once we implement the weights using realistic RRAM devices. Figure 1A shows a typical network topology used in the simulations. When training, all 60,000 MNIST training images were used; they were divided into training and verification sets in the ratio 3:1. All 10,000 test images were used to test the accuracy of the neural networks. A feed-forward neural network architecture was used with 784 input neurons (representing the pixel intensities of images of 28 × 28 pixel size) and 10 output neurons (representing 10 digits). Each of the hidden layers (typically two hidden layers, unless stated otherwise) consisted of 100 neurons. All of them used the sigmoid activation function, while the output layer used a softmax activation function and a cross-entropy error function was used in the learning process accordingly. The code was implemented in Python with functions realizing the sampling of modified PERT distribution imported from R.

For every architecture explored, 30 different base networks, that had been trained separately, were used in the simulations in order to produce average classification accuracy. In the case of deterministic discretisation, non-linear I/V and non-linear programming, each of the base networks with continuous weights was discretised only once because these simulations were not probabilistic in nature; thus, in the graphs summarizing those results, every data point represents an average of 30 accuracies. In the analysis of faulty devices, each discretised network was pruned or some of its devices were set to a specific conductance state 30 times for each of the proportions, thus, in the graphs summarizing those results, every data point represents an average of 900 accuracies. In the case of modified PERT disturbance, each discretised network was disturbed 20 times, thus, in the bar chart summarizing those results, every bar represents an average of 600 accuracies.

## 3. Results and Discussion

### 3.1. RRAM Experimental Results

**Figure 2:**
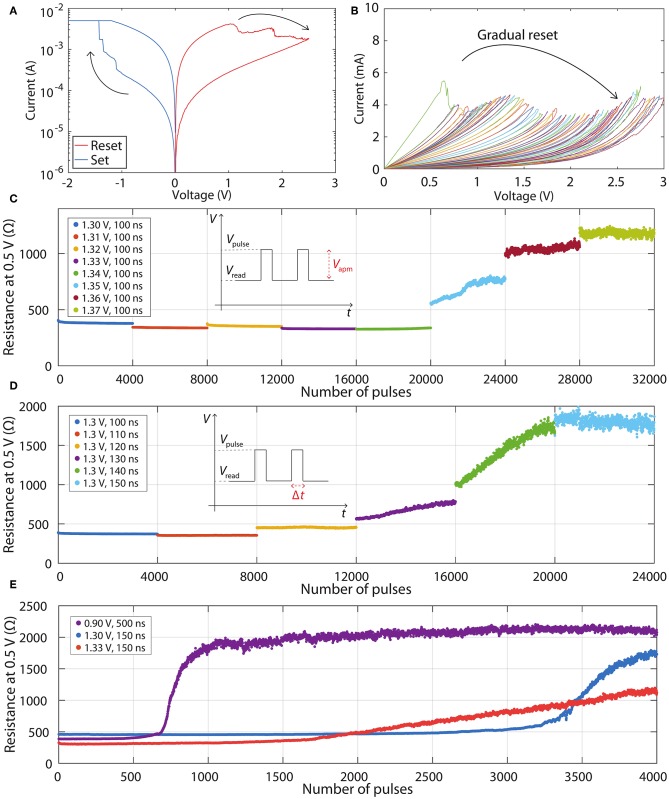
**(A)** Typical IV curves demonstrating resistance switching. **(B)** Reset voltage sweeps demonstrating gradual switching from LRS to HRS. **(C)** LTD characteristics in dependence of increasing pulse amplitude. **(D)** LTD characteristics in dependence of increasing pulse width. **(E)** Three different LTD characteristics obtained from the same RRAM device.

Figure 2 shows typical experimental results obtained from our SiO_*x*_ RRAM devices. Figure 2A demonstrates typical set and reset switching curves and two clearly defined resistance states—low resistance state (LRS) and high resistance state (HRS). More details of all switching characteristics, including retention, endurance, variability, as well as further information about the mechanism of the switching process and the microstructure of the SiO_*x*_ layers, can be found in our previous publications (Mehonic et al., 2015, 2017, 2018; Munde et al., 2017; Kenyon et al., 2019). In this paper, we focus on obtaining multiple intermediate resistance states, which are crucial for implementing programmable weights in neural networks. As seen in the voltage sweeps in Figure 2A, the reset process is typically gradual; in contrast the set process is abrupt—the current increase from the HRS to the LRS occurs at a single data point. Therefore, we obtain multiple resistance states by controlling the reset process. Achievability of many resistance states by progressively increasing the stop reset voltage in voltage sweeps is demonstrated in Figure 2B. Numerous different stable resistance states are obtained as devices switch incrementally from the LRS to the HRS.

However, practical and efficient programming of RRAM crossbars requires pulsed operation rather than voltage sweeps. In the past, many different programming schemes have been demonstrated, some of which employ voltage pulses with varying amplitudes and widths (Wang et al., 2016). However, these schemes increase the complexity of external drive circuitry, hence here we focus on identical voltage pulses. First, we set devices into the LRS typically with a short set pulse (-1.3 V, 100 ns). Then we analyse the gradual reset programming with identical voltage pulses. After each programming pulse, the state is probed by a reading pulse (0.5 V, 1 μs). We found that the nature of gradual resetting is highly dependent on both voltage and pulse width. We fine-tune the voltage with a fixed width, or the pulse width with a fixed voltage. This is demonstrated in Figures 2C,D, respectively. We find that varying either of these two parameters even by relatively small amounts changes dramatically the gradual switching characteristics. Figure 2C shows eight different characteristics. Every characteristic consists of 4000 voltage pulses with a fixed width of 100 ns and an increase of the voltage of 10 mV between successive characteristics. No significant change in resistance is observed until the amplitude is increased to 1.35 V, after which an abrupt resistance jump is followed by gradual modulation. A similar effect is observed by fixing the amplitude of the pulse to 1.3 V and varying the pulse width from 100 ns in increments of 10 ns. Again, no significant change is observed until the pulse width reaches 130 ns, after which a gradual resistance increase is observed. The rate of the resistance increase is controlled by changing pulse widths.

These programming curves are typically called depression or long-term depression (LTD) curves. For programming simplicity, linear curves are preferred. Unfortunately, this is typically not the case if identical voltage pulses are used: the curves often have exponential/logarithmic shapes. However, it is important to realize that the shape of these curves is dependent not only on the particular RRAM devices but also on the fine-tuning of the programming pulses. Figure 2E shows three very different LTD curves obtained by using the same device but different amplitudes and widths of programming pulses.

It is interesting to observe that it is possible to affect both the ratios between the maximum and minimum resistances, as well as the shapes of the curves, by fine-tuning the amplitudes and widths of the pulses. The purple and blue curves are highly non-linear, while the red curve is closer to preferred linear response. Here, we do not focus on optimizing the programming scheme, but we speculate that programming can be finely tuned to exhibit a linear response.

### 3.2. Analysis of the Effect of RRAM Non-idealities on Inference Accuracy

#### 3.2.1. Weight Mapping Onto RRAM Devices

Depending on the type of RRAM devices used, as well as programming schemes utilized, it is important to understand the effect of weight mapping and discretisation on the ANN performance. There are two important points to consider when representing synaptic weights using discrete conductance states: the choice of the discrete levels that continuous weights will be mapped onto, and how the continuous weights between two discrete levels would be mapped onto one or the other level. Regarding the first point, we explore proportional mapping scheme which makes the conductances proportional to the synaptic weights. This is the simplest approach being considered by others (Yu, 2018) because it minimizes the complexity of required circuitry for mapping. When discrete levels are determined, we map continuous weights onto those levels by rounding, i.e., mapping them to the closest discrete level. Randomized rounding (Muller and Indiveri, 2015) could be employed but it is not as effective with *ex-situ* learning as simple rounding is.

**Figure 3:**
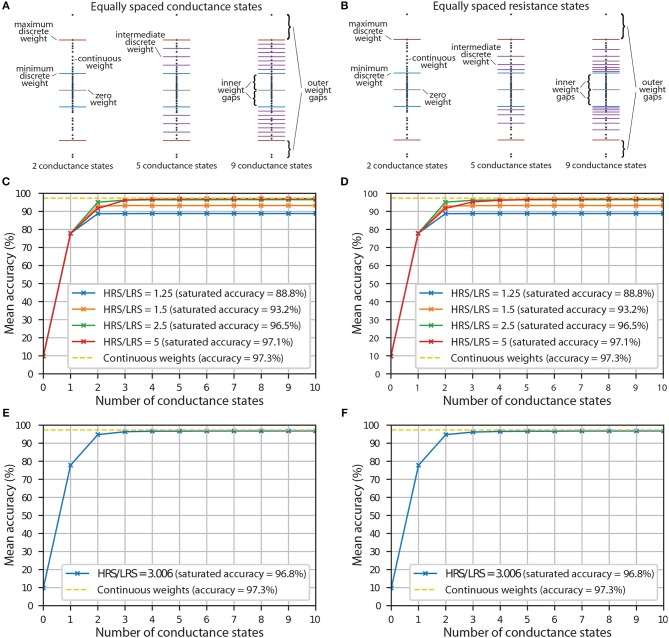
**(A)** An illustration showing synaptic weights represented by equally spaced conductance states. **(B)** An illustration showing synaptic weights represented by conductance states corresponding to equally spaced resistance states. **(C)** The accuracy of a neural network with equally spaced conductance states being used as representations of discrete synaptic weights. Results for different HRS/LRS ratios are shown. **(D)** The accuracy of a neural network with conductance states corresponding to equally spaced resistance states being used as representations of discrete synaptic weights. Results for different HRS/LRS ratios are shown. **(E)** The accuracy of a neural network with equally spaced conductance states being used as representations of discrete synaptic weights. Experimental HRS/LRS ratio was used. **(F)** The accuracy of a neural network with conductance states corresponding to equally spaced resistance states being used as representations of discrete synaptic weights. Experimental HRS/LRS ratio was used.

In proportional mapping, it is important to decide what the maximum discrete weight, *w*_max (discrete)_, in each synaptic layer will be. SiO_*x*_ RRAM devices that do not electroform have a resistance of >1011 Ω (Mehonic et al., 2012), which is a good approximation to an open connection. Thus, the zero weight, *w*_0_ = 0, will be implemented by simply not electroforming the devices, but the minimum nonzero discrete weight, *w*_min (discrete)_, will be equal to $\frac{w_{\text{max }\left(\text{discrete}\right)}}{\text{HRS}/\text{LRS}}$, where HRS/LRS is the ratio between highest achievable conductance, *G*_max_, and lowest achievable conductance, *G*_min_, of electroformed RRAM devices. Because of this, there will exist a region between *w*_0_ and *w*_min (discrete)_, where the continuous weights will not be represented perfectly, no matter how many intermediate states are added between *w*_min (discrete)_ and *w*_max (discrete)_; we call this region an inner weight gap. To reduce the inner weight gap in a given device we can choose a smaller *w*_max (discrete)_, but that creates an outer weight gap above *w*_max (discrete)_ (see Figures 3A,B). In practice, with the ANNs that we trained, most of the continuous weights are concentrated around the zero weight, with a small number of weights forming thin tails at both ends (see Figure S1). Thus instead of setting *w*_max (discrete)_ equal to the continuous weight, *w*_max_, that has the largest absolute value, we find that it is more appropriate to exclude a small proportion, *p*_L_, of weights with the largest absolute values. This technique is summarized in Algorithm 1 (Supplementary Material). With our ANNs, we find that the most appropriate value of *p*_L_ is ~0.015, i.e., we exclude a total of 1.5% of the weights at the tails when choosing *w*_max (discrete)_. This value of *p*_L_ was used in all of the simulations involving discretisation in this analysis. For significantly different HRS/LRS ratios or very few discrete states, different value of *p*_L_ might be more appropriate.

To avoid the weight gaps altogether, one could employ a different mapping scheme that not only scales the weights non-linearly but also shifts them by a certain amount (Tarkov, 2015). However, if neuronal circuits were employed, this approach would result in needlessly complex circuitry required for these non-linear transformations which would defeat the purpose of having a simple architecture that the crossbar arrays can provide. The other approach is to use a difference of the conductances of two memristors as an effective conductance and representation of the synaptic weight. This method was used to achieve state-of-the-art accuracy by augmenting PCM devices with CMOS circuitry to deal with non-idealities (Ambrogio et al., 2018). This mapping scheme contributes to the circuit complexity because two, rather than one, memristors per every synaptic weight have to be finely tuned. Therefore, we focus on proportional mapping and fine-tuning of only one memristor per every synaptic weight in this analysis.

To implement negative weights using conductances, two crossbar arrays per synaptic layer are typically necessary (Hu et al., 2012)—one for positive and one for negative weights, as shown in Figure 1B. If a weight is negative, it would be represented by a dedicated memristor in the crossbar of negative weights (where negative input voltages would be used). The corresponding memristor in the crossbar of positive weights (where positive input voltages would be used), would be unelectroformed, resulting in its negligible effect on the total output current (which is computed by adding corresponding currents from the two crossbars). The implementation of a positive weight would be opposite to that of a negative weight. Thus, taking into account the zero level, having *N*_G_ conductance states (of electroformed devices) would result in 2*N*_G_ + 1 discrete levels.

With our RRAM devices, we can achieve continuous modulation of conductance by employing voltage pulses. But if a hypothetical device only has a finite number of intermediate conductance states available, the performance of the hardware implementation of an ANN might depend on how these states are spaced. Of course, if the device exhibits discrete conductance states, it is usually not possible to choose how those states are spaced. However, some patterns of how the states are distributed might be more probable than others. One possibility that we explore is an equal spacing between conductance states. This is demonstrated in Figure 3A. As mentioned earlier, even with an infinite number of conductance states, the continuous weights in weight gap regions will not be represented perfectly. The inner weight gaps are reduced by increasing HRS/LRS ratio. This will be an important metric in our simulations.

Another possibility that we analyse is equal spacing between resistance states. This is illustrated in Figure 3B. The main difference between this scheme and the scheme which used equal spacing between conductance levels is that in this scheme, we have a higher density of discrete levels near *w*_min (discrete)_—a result of reciprocal relationship between conductance and resistance. This, in theory, can be disadvantageous, because if we exclude the thin tails at both ends, the weights are usually distributed relatively uniformly in the synaptic layers (apart from the weights in the last synaptic layer, see Figure S1C) of ANNs that we used. In practice, we find that choice of the scheme has little effect most of the time. This suggests that it is most important to have enough discrete states, while the exact spacing between each is not that important, as long as they are relatively uniformly distributed, i.e., not concentrated around a single value.

#### 3.2.2. HRS/LRS Ratio in Proportional Mapping

As discussed earlier, the inner weight gaps, which are partly determined by the HRS/LRS ratio, can dramatically affect the network performance in both scenarios. It is essential to understand how the HRS/LRS ratio influences the accuracy of a neural network with both low and high number of conductance states.

The results for implementations with both equally spaced conductance states and equally spaced resistance states are shown in Figures 3C,D, respectively. Zero conductance states correspond to a scenario when all the devices are unelectroformed, and thus all the weights are set to zero; this results in random chance that the ANN will guess the class correctly out of the 10 available, i.e., mean accuracy of ~10%. A single conductance state corresponds to a scenario with some of the devices being unelectroformed, and some electroformed and set to LRS, i.e., 3 discrete levels in total because the positive weights are reflected around the zero weight using the second RRAM array, as mentioned earlier. Because at this stage none of the devices are switched to HRS, HRS/LRS ratio does not have an effect and all the curves have the same accuracy at that point. Only at 2 conductance states, when some devices are switched to LRS, do we start to see the differences in accuracy for different HRS/LRS ratios. At 3 conductance states, we, in theory, should start noticing the differences in accuracy between equally spaced conductance and resistance states because intermediate discrete levels are introduced. In practice, we see little difference between the two schemes, except for the general trend that it takes longer for the accuracy to saturate with equally spaced resistance states if HRS/LRS ratio is high. This is usually not visible by the naked eye, though one might notice that when HRS/LRS = 5, accuracy with 3 equally spaced conductance states is slightly larger than accuracy with 3 equally spaced resistance states. Both plots show that the higher the HRS/LRS ratio is, the higher the maximum achievable accuracy by the corresponding ANN is. We define saturation to happen when an ANN with a certain number of conductance states produces the same accuracy (correct to the tenths digit) as an ANN with an infinite number of intermediate conductance states. We note that the accuracy saturates at different number of conductance states depending on the HRS/LRS ratio. For example, when HRS/LRS = 1.5, accuracy saturates at 3 conductance states with both schemes, while when HRS/LRS = 5, accuracy saturates at 8 equally spaced conductance states and at 12 equally spaced resistance states. Also, if RRAM devices have a very large HRS/LRS ratio and enough conductance states, it might sometimes be advantageous to decrease the outer weight gap by decreasing *p*_L_ (and thus increasing *w*_max (discrete)_).

Experimental results from the linear region of the red curve in Figure 2E were used to extract HRS/LRS ratio of 3.006. The effect of the number of conductance states on accuracy (with experimental HRS/LRS ratio) either by using equal spacing between conductance or resistance states, is shown in Figures 3E,F, respectively. Both of them yield almost identical results. In both plots, the accuracy of discretised networks saturates at around 96.8% at 6 equally spaced conductance states and at 7 equally spaced resistance states. This saturation is the result of the weight gaps mentioned earlier—a number of continuous weights will not be represented perfectly no matter how many conductance states are added.

Even though Figure 2E demonstrates almost continuous resistance changes, discretisation simulations exploring the effect of a small number of states are nevertheless valuable to allow for other RRAM devices that might exhibit only a finite number of discrete states.

#### 3.2.3. Faulty Devices

RRAM devices might sometimes not work the way they were designed to and this could manifest itself in different ways. One of the most common problems is devices that cannot electroform. Because of the high resistance of the pristine state, this (in the context of proportional mapping) is effectively the same as having a zero weight. Of course, when ANNs are discretised, some of the synaptic weights are supposed to be zero. However, one cannot guarantee that the devices responsible for implementing nonzero weights will electroform, thus a drop in accuracy is expected if these devices are indeed not capable of electroforming. We will refer to the proportion of the devices that are capable of electroforming as yield.

**Figure 4:**
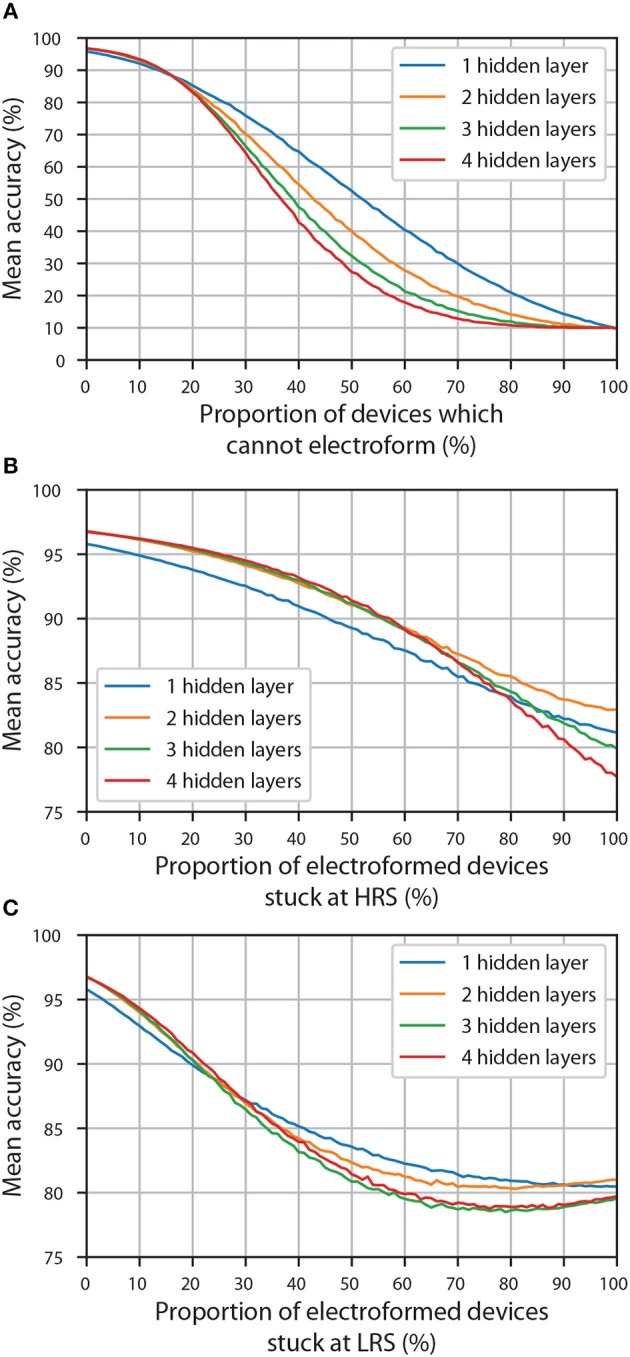
The accuracy of ANNs (with different number of hidden layers) discretised using 10 equally spaced conductance states and physically implemented using faulty devices. **(A)** The effect of having devices which cannot electroform. **(B)** The effect of having electroformed devices which are stuck at HRS. **(C)** The effect of having electroformed devices which are stuck at LRS.

Figure 4A shows the dependence of classification accuracy of discretised ANNs with different proportions of their weights pruned (set to zero); results for different number of hidden layers are shown. The starting accuracy (referring to the accuracy with all the devices working perfectly) influences the robustness to pruning: the higher the starting accuracy, the more robust network is to pruning (this is explained in more detail in the Supplementary Material). Despite of ANNs containing one hidden layer having the lowest mean starting accuracy (which is not necessarily a universal result, but only a consequence of our training and discretisation procedures), their accuracy eventually becomes higher than that of any other architecture as more of the weights are pruned. The other three architectures, which have a very similar starting accuracy, follow the same trend: the fewer hidden layers, the more robust ANNs are to pruning. This is significant as it shows that the yield of devices can determine the most appropriate network architecture that should be utilized when using crossbar arrays. In the case of the ANNs we trained, a discretised network with 2 hidden layers, each containing 100 neurons, might perform better than a network with one hidden layer if all of the devices responsible for realization of nonzero weights have electroformed, but for a non-ideal yield of RRAM devices (e.g., 80%), it might be preferable to choose an ANN with one hidden layer.

We speculate that this decrease in robustness to pruning in *ex-situ* training, where networks cannot easily adapt to non-idealities, is a result of decreased parallelism of an ANN. The reason for an ANN robustness in general can be explained by its parallel nature—each neuron (or synapse) is responsible only for a small portion of the network's operation; not a single one of them is critical to the performance. However, because layers are added in series, rather than in parallel, pruned synapse in a deep neural network will have a cascading effect over all the hidden layers. We did not find enough evidence that increasing the number of neurons in each hidden layer can increase the robustness to pruning; it is probable that the increased parallelism of an ANN (produced by extra neurons in each hidden layer) is canceled out by pruning—more synapses are present, but also more of them are pruned because we are removing the same *proportion* every time.

Although less relevant to *ex-situ* training, we investigate the effect of electroformed devices getting stuck at either HRS or LRS state responsible for the realization of *w*_min (discrete)_ and *w*_max (discrete)_, respectively.

The effect of devices getting stuck at HRS is shown in Figure 4B and is clearly less extreme that that of pruning. The main reason for that is that even when all of the electroformed devices are stuck at HRS, not all of the information is lost: one is still left with 3 discrete weight levels (zero weight realized using unelectroformed devices, as well as one positive and one negative weight realized using stuck electroformed devices and two crossbar arrays) and thus an accuracy in the range 78-83%, rather than ~10% which is the case when all the weights are set to zero (Figure 4A). From these findings alone, it is difficult to make any meaningful conclusion about the effect of number of hidden layers. Though if one excludes ANNs with one hidden layer (that have a visibly lower mean starting accuracy), one could claim that the accuracy of ANNs with fewer hidden layers eventually becomes higher. Of course, this becomes apparent only when ~70% of the electroformed devices become stuck at HRS which is unlikely to happen in practice.

The effect of devices getting stuck at LRS is shown in Figure 4C; the decrease in accuracy is more sudden initially than in the case of devices getting stuck at HRS. In this case, it is even more difficult to interpret the effect of the number of hidden layers because some of the curves intersect each other more than once. An interesting effect is observed when accuracy curves with two, three and four hidden layers have their local minima not at 100% of the electroformed devices being stuck at LRS, but at a smaller proportion. We speculate that this is due to the fact that LRS is used to implement *w*_max (discrete)_. During the discretisation stage, synapses that are the most significant were already set to *w*_max (discrete)_ (or −*w*_max (discrete)_) and thus were dominant, especially in the last synaptic layer. When some of the synapses which had lower weights were set to *w*_max (discrete)_, they became dominant too, thus decreasing the relative importance of the synapses that were supposed to have a weight with the highest absolute value. At some point so many of the synapses will be randomly assigned high weights that any column of RRAM devices (see Figure 1B) which will not have enough devices at LRS will not be able to produce enough current to have any significant effect. At that point, it would be better if all of the electroformed devices were stuck at LRS, thus all having equal importance.

#### 3.2.4. I/V Non-linearity

**Figure 5:**
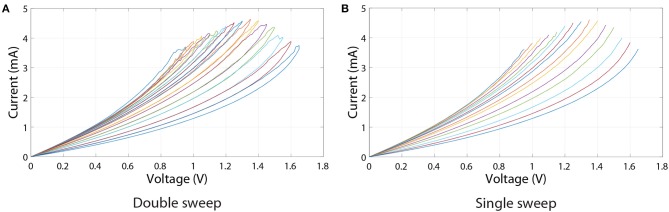
Voltage sweeps of resistance states lying in the linear programming pulses region of the red curve in Figure 2E. **(A)** Double sweep. **(B)** Single sweep.

Another important non-ideality of RRAM devices is non-linearity of I/V characteristics. Because inputs to every synaptic layer would be represented by voltage signals, this non-linearity can further affect the accuracy of the ANN, unless the data set inputs are binary and binary neurons are used (neither of which is true in our case). Here, we analyse I/V curves corresponding to the operational regions from the experimental results shown in Figure 2E (red curve). Note that a significant resistance change is not observed throughout the full range of voltage pulses. In order to only use voltage sweeps' results from Figure 2B that correspond to operational region shown in Figure 2E (red curve), which is between 382 and 1150 Ω, only the resistance states within this resistance range were used. Voltage sweeps corresponding to these 15 states are separately shown in Figure 5A.

To quantify the effect of I/V non-linearities on RRAM ANN performance, ANNs were firstly discretised using the above-mentioned 15 resistance states (resulting in 31 different synaptic weights). Then the bottom branches of I/V curves in Figure 5A (shown separately in Figure 5B) were used as a lookup table to compute the currents flowing out of the crossbar array. Of course, before computing the currents, one needs to decide what inputs correspond to what voltages. In the software model, inputs to every synaptic layer are within the range [0, 1]. In order to convert the software inputs to voltage (in volts), we multiply inputs by an input scaling factor, *k*. This factor is also used to convert the resulting currents to software outputs. The reset voltage of the leftmost curve in Figure 5 is 0.95 V, meaning that any voltage value above that will not have a corresponding current output. Thus, *k*, in our case, can be at maximum equal to 0.95.

**Figure 6:**
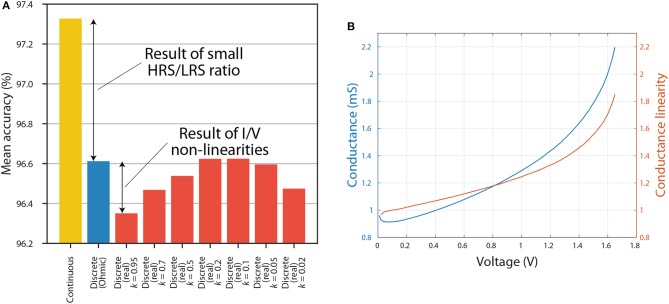
**(A)** The inference accuracy when using continuous weights (yellow), when using discrete weights without taking into account I/V non-linearities (blue) and when using discrete weights by taking into account I/V non-linearities with different values of *k* (red). **(B)** Dependence of conductance and conductance linearity on voltage. The figure shows results for the most non-linear I/V curve from Figure 5.

Results showing the effect of varying *k* on inference accuracy are presented in Figure 6A. These results suggest that the choice of *k* can have a noticeable effect on inference accuracy. For example, choosing *k* = 0.1 or *k* = 0.2 can lead to an accuracy that is as good as that of a discretised ANN which treats RRAM devices as Ohmic resistors. That is partly because, as can be seen in Figure 5B, the I/V curves of all resistance states are highly linear at low voltages. However, if *k* is decreased even further, we observe decrease in accuracy. This might be due to a current offset—nonzero current at 0 V. In RRAM crossbar array applications it is more important that the ratio between current and voltage, rather than the slope of I/V curve, stays constant because crossbar multiply-accumulate (MAC) operations rely on the idea that *I* = *GV*, where *I* is current, *G* is conductance and *V* is voltage. Another factor that has to be considered is how the conductance of a certain state is measured. In our case it was measured using 0.5 V reading pulses, and these values were used to discretise the ANNs. However, non-linear I/V curves result in a dependence of conductance on voltage, thus the value measured using a reading pulse is representative only of a small portion of an I/V curve. The interplay between this and the overall linearity of I/V curve determines how *k* influences accuracy in Figure 6A. Furthermore, the accuracy of Ohmic discrete ANNs using proportional mapping scheme is fundamentally limited by the HRS/LRS ratio, which in this case is set to be relatively low at 2.644 (from the 15 I/V curves).

Figure 6B shows the dependence of conductance and conductance linearity on voltage. As mentioned before, conductance will be defined as the ratio between current and voltage, rather than the slope of the I/V curve. Here we introduce the metric of conductance linearity (CL) described in reference (Sung et al., 2018). CL at a certain voltage *V*_R_ is defined as the conductance at that voltage divided by conductance at 0.5*V*_R_; the closer this ratio is to 1, the more linear conductance is considered to be. Figure 6B reiterates the point that I/V curves become more non-linear at high voltages.

#### 3.2.5. Non-linear Programming With Voltage Pulses

**Figure 7:**
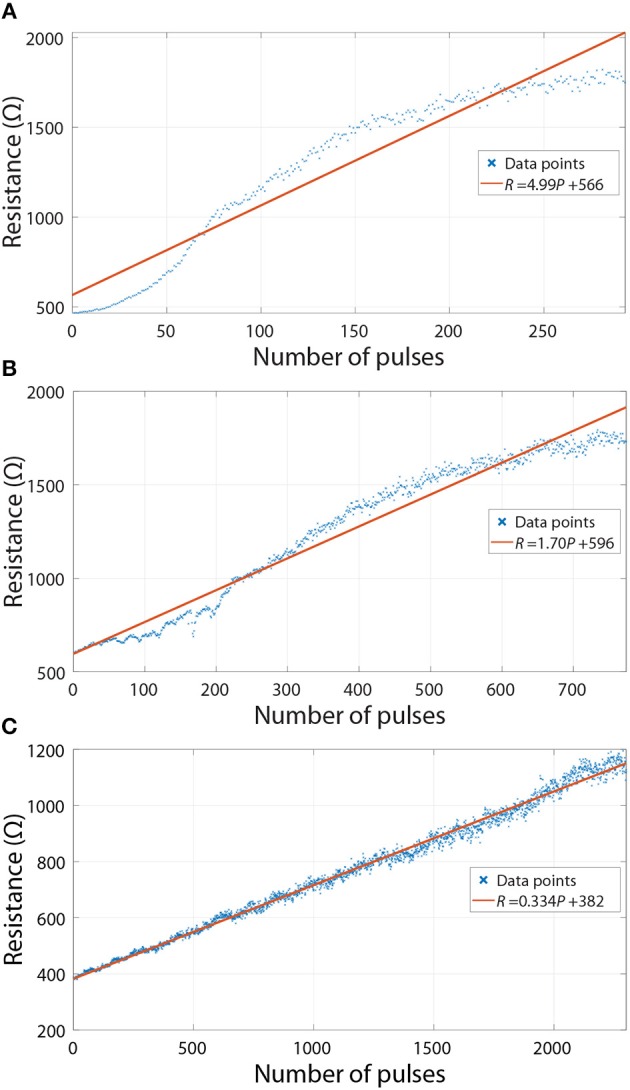
Experimental results from real RRAM devices used in the simulations—resistance changes of devices when applying voltage pulses. Linear fits are also shown in the plots. **(A)** Purple curve in Figure 2E. **(B)** Blue curve in Figure 2E. **(C)** Red curve in Figure 2E.

Although the weights are fixed during the inference phase, we analyse how the deviations from linear programming during the initial setting of weights (as part of *ex-situ* training) affect the accuracy. Linear programming is preferred as it leads to reduced circuit complexity. To understand the effects of deviations from a model that relates the number of programming pulses to resistance changes, the experimental results in Figure 7 were considered. These are operational regions previously shown in Figure 2E (we do not consider the regions where device resistance is not significantly changed by voltage pulses). In the most straightforward programming scheme, the ANNs would be discretised and the crossbars would be programmed using a linear model (fits for such models are also shown in Figure 7) without applying reading pulses to confirm the resistance values. Because the model is not perfect, it would result in the actual resistances being different from the ones used in the discretisation of the network. This affects the performance of the ANN.

**Figure 8:**
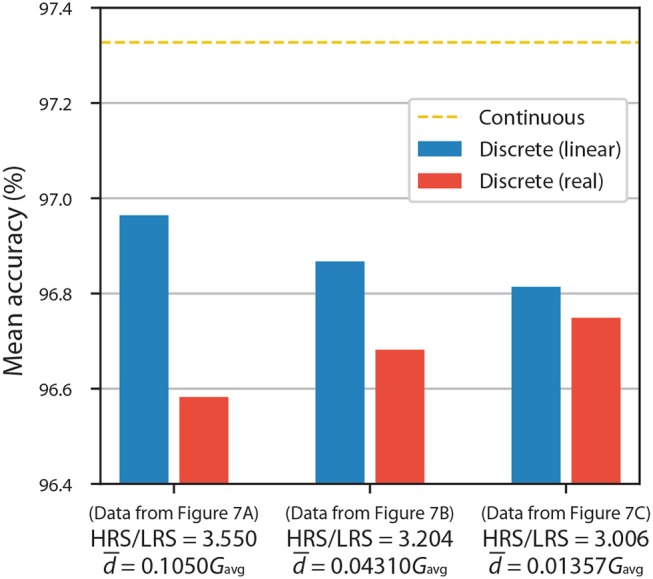
Bar chart of accuracies of ANNs with 10 equally spaced conductance states being used as representations of discrete synaptic weights. Figure shows results for different pulsing experiments from Figure 7. Blue columns assume linear resistance changes, while red columns use real experimental data.

To quantify the extent of this effect, we firstly discretized the networks using linear fits (see Figure 7) and then used the experimental resistance values to investigate differences in accuracy. The results for ANNs with 10 equally spaced conductance states are shown in Figure 8. Blue columns, which assume linearity, reiterate that having a larger HRS/LRS ratio results in higher accuracy. When the deviations from the model are taken into account, we can observe moderate decreases in accuracy for highly non-linear experimental results (Figures 7A,B), as is indicated by the red columns in Figure 8, but even they are limited to a maximum of ~0.4% decrease (with respect to accuracy of linear discrete model) in the worst-case scenario.

It is important to note that the $\bar{d}/G_{\text{avg}}$ ratios (denoting average absolute deviation, $\bar{d}$, of conductance divided by average conductance, $G_{\text{avg}}=\frac{G_{\text{min}}+G_{\text{max}}}{2}$) presented in Figure 8 are not perfect metrics for quantifying these deviations. They only capture the magnitudes of deviations but not their global behavior. This metric does not take into account wave-like characteristics of the curves in Figure 7, which result in some resistance regions being disturbed more than the others; this leads to a greater decrease in the accuracy of ANNs than if the disturbances were independent from the value of resistance. Therefore, two different experiments with the same HRS/LRS and $\bar{d}/G_{\text{avg}}$ ratios will generally not result in the same accuracy.

#### 3.2.6. Device-to-Device Variability

One of the advantages of *ex-situ* learning is that the conductance of memristor has to be precisely set only once, since the devices will not be switched during the operation of the physical ANN. Here, two points have to be considered: (1) how straightforward it is to set those conductances precisely over all programmed devices, and (2) whether the conductance will change over time.

The first point was partly explored in sub-subsection 3.2.5. However, it was built on the assumption that we can easily identify the linear range of operation of the device, and that this range will be the same for all the devices. This is unlikely to be the case in practice; it might be easier to set the values around the midpoint of *G*_min_ and *G*_max_, but the conductances near *G*_min_ or *G*_max_ might be more difficult to achieve, as can be seen from Figure 2E: the curves flatten out near *G*_min_ and near *G*_max_ (or equivalently near *R*_max_ and near *R*_min_, as seen in the plot). If one wanted to set the conductance close to *G*_min_, it is likely that the conductance achieved would be higher than desired. On the other hand, if one wanted to set the conductance close to *G*_max_, it is likely that the conductance achieved would be lower than desired. This is typical for most RRAM devices as they exhibit a non-linear response to voltage pulses.

The second point is related to the retention of the devices. Ideally, the conductance of the device would be constant. In practice, the conductance might change slightly over time; this is the effect of weak filaments that can self-dissolute after some time. Similarly, in the case of random telegraph noise (RTN), device conductance can abruptly shift between two or more states over time.

These and other types of device-to-device variability are difficult to model realistically, and the models might differ in major ways depending on the type of RRAM devices used. However, we aim to model variations between individual devices at least qualitatively by considering the probabilities of devices' conductances deviating from desired values by certain amounts in certain directions. This could represent the effect of non-ideal programming from device to device or time-induced drift/shift from the desired values (as in the case of RTN). Deviations can be modeled by sampling from a certain probability distribution, but given that a physical characteristic (conductance) of the electroformed devices is being modeled and that we want to reflect the qualitative features of variability described above, it is important to have certain constraints for the probability density function (PDF) of such distribution:

1. Electroformed devices should always have a conductance which is between minimum conductance, *G*_min_, and maximum conductance, *G*_max_, i.e., the probability of the conductance being disturbed outside this range should be zero—PDF should be defined on a bounded interval [*G*_min_, *G*_max_].
2. If the conductance is closer to *G*_min_ than it is to *G*_max_, it should have a higher probability of deviating toward *G*_max_ than toward *G*_min_, and vice versa.
3. It should be possible to define the mode of a PDF; in this way we could use the desired conductance as the mode.
4. It should be possible to define the extent of deviation; in this way we could reflect different degrees of uncertainty.

One of the PDFs satisfying all the requirements is the modified PERT distribution. It is defined on a bounded interval [*a, b*], and is characterized by its mode, *m*, and shape parameter, γ (Vose, 2008). It becomes skewed if it is too close to one of the endpoints and one can also change its variance, σ^2^, by adjusting γ. However, because variance is defined in terms of average of a PDF—and not mode which we are interested in—it is better to choose a different metric of uncertainty: we use average absolute deviation, $\bar{d}$, of conductance from the mode; this number gives the average absolute deviation of disturbed conductance, *G*_new_, of electroformed device from the original conductance, *m*. There is no analytic expression for γ in terms of $\bar{d}$, thus one needs to compute γ numerically.

We stress that this kind of disturbance will be continuous in nature and only applied to electroformed devices because the conductance (and thus its variability) of unelectroformed devices is orders of magnitude lower. Also, the conductance is most likely to be disturbed by a small amount (because mode, *m*, represents the original conductance), but there is a small probability of it being disturbed by a large amount. After disturbance, the electroformed devices are no longer in discrete conductance states, though if the disturbance is not too large, the peaks of the conductance distribution of all the devices will be visible at the positions of the original discrete conductance states.

**Figure 9:**
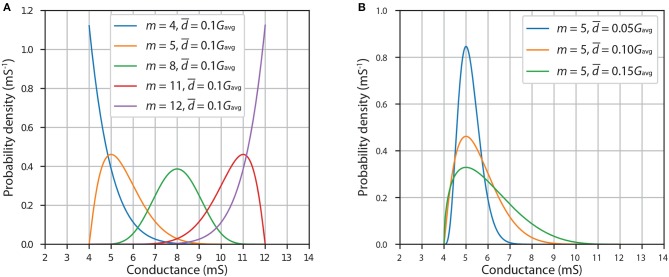
Probability density functions of modified PERT distribution with lower limit 4 mS and upper limit 12 mS. **(A)** Effect of changing mode, *m*. **(B)** Effect of changing average absolute deviation, $\bar{d}$.

Figure 9 shows example modified PERT PDFs for a device with *G*_min_ = 4 mS and *G*_max_ = 12 mS (and thus $G_{\text{avg}}=\frac{G_{\text{min}}+G_{\text{max}}}{2}=\text{8}\;\text{mS}$). Figure 9A shows the effect of changing the mode, *m*, of the distribution, which represents the value of desired conductance, while keeping the average absolute deviation, $\bar{d}$, constant at 0.1*G*_avg_ = 0.8 mS. If *m* = 8 mS, then the shape of the PDF of this distribution most resembles the PDF of normal distribution with mean 8 mS. However, it is not exactly the same shape because in this case Pr(*G*_new_ < 4) = Pr(*G*_new_>12) = 0, unlike in normal distribution where the tails extend infinitely far in both sides. If *m* is close to one of the endpoints, the PDF becomes noticeably skewed toward the other endpoint. Because 5 mS is the same distance from the endpoint *G*_min_ as 11 mS is from the endpoint *G*_max_, the PDFs with *m* = 5 mS and *m* = 11 mS are symmetrical. When *m* lies at one of the endpoints, the PDF has only one tail extending toward the other endpoint. Figure 9B shows the effect of changing the average absolute deviation, $\bar{d}$, while keeping the mode, *m*, constant at 5 mS. We note that the lower the deviation, the more localized and less skewed the PDF becomes.

**Figure 10:**
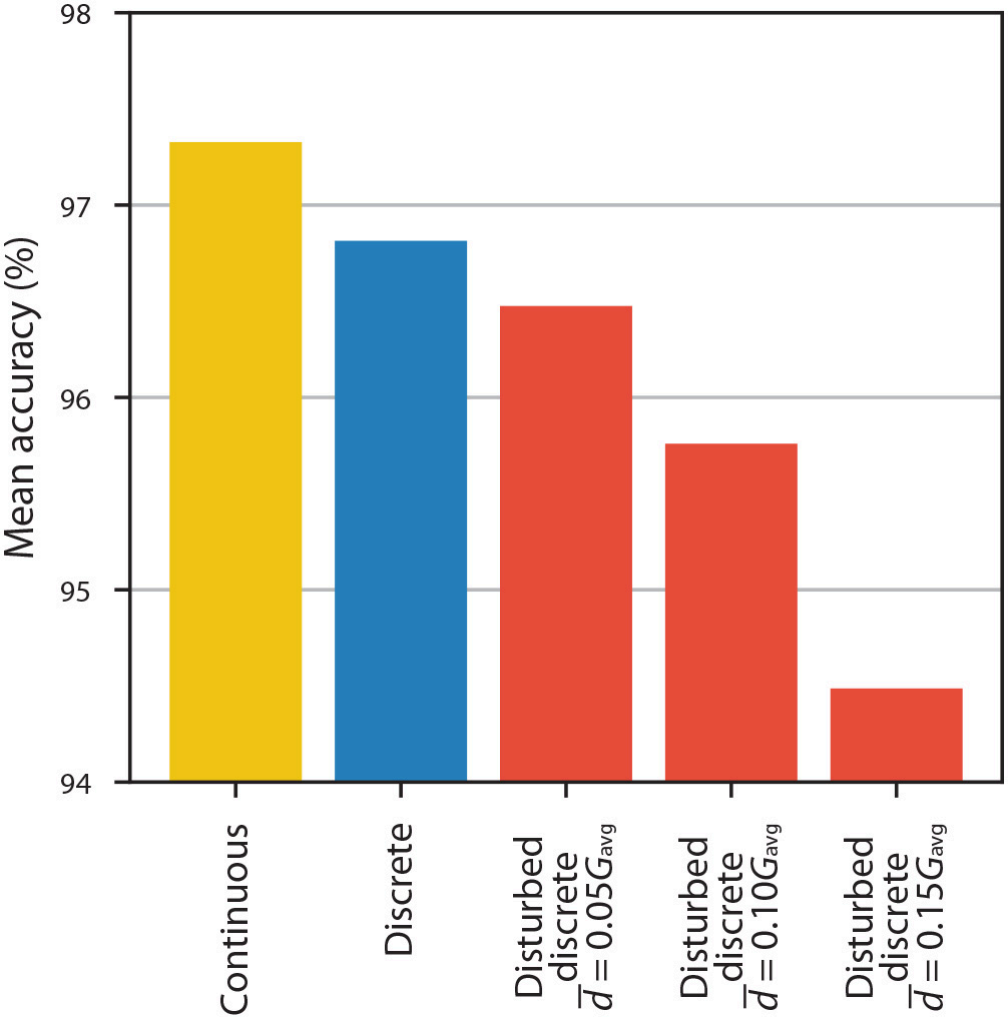
The accuracy of ANNs with continuous weights (yellow), with discrete weights obtained from 10 equally spaced conductance states and HRS/LRS = 3.006 (blue), and with the same discrete weights that were disturbed using modified PERT distribution (red).

Results for ANNs with HRS/LRS = 3.006 and 10 equally spaced conductance states being disturbed using this model are presented in Figure 10. We observe that if the average absolute deviation of conductance is equal to 5% of the average conductance, then the decrease in accuracy is relatively small-~0.3%-smaller than the difference between accuracy with continuous weights and accuracy with undisturbed discrete weights. Disturbances of larger magnitude result in more significant drops in accuracy: ~1.1% with $\bar{d}=0.10G_{\text{avg}}$ and ~2.3% with $\bar{d}=0.15G_{\text{avg}}$.

## 4. Conclusion

We have discussed a number of non-idealities of RRAM devices and their effects on inference accuracy when the weights in ANNs are implemented using realistic RRAM devices. First, we presented experimental results obtained from SiO_*x*_ RRAM devices and discussed important experimental considerations to achieve the best device properties. Our inference accuracy analysis includes: (1) Weight mapping onto RRAM devices; (2) HRS/LRS ratio; (3) Faulty devices; (4) I/V non-linearity; (5) Non-linear programming with voltage pulses; (6) Device-to-device variability.

Some of these factors have a more significant impact on accuracy than do others. For the particular demonstration application that we consider (recognition of handwritten digits using MNIST data), we find that the HRS/LRS resistance ratio has a significant impact on accuracy when using proportional mapping scheme. However, even a modest ratio of 5 provides accuracy of ~97.1% which is close to the accuracy of ~97.3% with continuous weights. For our experimental HRS/LRS ratio of 3.006, we find that mean accuracy of ~96.8% can be achieved. There is more than one way of how discrete states can be separated in realistic RRAM devices, but if they are relatively uniformly distributed, we find that accuracy saturates at 7 or fewer conductance states with HRS/LRS = 3.006. Our experimental results show that it is possible to achieve almost continuous modification of resistances, but we stress that the accuracy is much more affected by HRS/LRS resistance ratio. The effect of device yield depends on the particular implementation of the ANN. However, for most implementations, a drop in accuracy, with few percent of the devices not being able to electroform, is tolerable. The effect of electroformed devices being stuck at one of the states is even less detrimental. We analyse our experimental results and demonstrate that the negative effect of small non-linearities in I/V curves can be eliminated by choosing appropriate voltage range. Although the programming of the weights is less relevant for the inference phase, we discuss and analyse the effects of non-linear programming with voltage pulses. Finally, we model device-to-device variations in electroformed devices and show that small deviations ($\bar{d}=0.05G_{\text{avg}}$) from the desired conductance can have a smaller negative effect to classification accuracy than discretisation, although larger deviations ($\bar{d}=0.10G_{\text{avg}}$ and $\bar{d}=0.15G_{\text{avg}}$) can have a significant negative impact.

In conclusion, we demonstrate that RRAM devices with non-optimized switching properties could still be used for the implementation of weights in physical ANNs, particularly during the inference phase. It is essential to consider various device properties in the context of the particular application. This will inform best programming procedures and optimal trade-offs between the complexity of programming and inference accuracy.

## Data Availability

All datasets generated for this study are included in the manuscript and/or the Supplementary Files.

## Author Contributions

AM designed the study, performed the electrical characterization of devices, interpreted the data, and wrote the first draft of the manuscript. DJ designed the study, performed the simulations, interpreted the data, and wrote the first draft of the manuscript. WN fabricated the tested devices. MB contributed to the interpretation of the data. AK oversaw the work and assisted in the writing of the paper. All authors contributed to the manuscript.

### Conflict of Interest Statement

The authors declare that the research was conducted in the absence of any commercial or financial relationships that could be construed as a potential conflict of interest.

## References

[B1] Ambrogio S. Narayanan P. Tsai H. Shelby R. M. Boybat I. Nolfo C. D.. (2018). Equivalent-accuracy accelerated neural-network training using analogue memory. Nature 558, 60–67. 10.1038/s41586-018-0180-5 29875487

[B2] Burr G. Shelby R. Sidler S. Nolfo C. D. Jang J. Boybat I. (2015). Experimental demonstration and tolerancing of a large-scale neural network (165,000 synapses), using phase-change memory as the synaptic weight element. IEEE Trans. Electron Devices 62, 3498–3507. 10.1109/TED.2015.2439635

[B3] Chai Z. Zhang W. Freitas P. Hatem F. Zhang J. F. Marsland J. (2018). The over-reset phenomenon in Ta_2_O_5_ RRAM device investigated by the RTN-based defect probing technique. IEEE Electron Device Lett. 39, 955–958. 10.1109/LED.2018.2833149

[B4] Chang T. Jo S. H. Lu W. (2011). Short-term memory to long-term memory transition in a nanoscale memristor. ACS Nano 5, 7669–7676. 10.1021/nn202983n 21861506

[B5] Chang Y. F. Fowler B. Chen Y. C. Zhou F. Pan C. H. Chang T. C.. (2016). Demonstration of synaptic behaviors and resistive switching characterizations by proton exchange reactions in silicon oxide. Sci. Rep. 6:1. 10.1038/srep21268 26880381 PMC4754682

[B6] Gokmen T. Vlasov Y. (2016). Acceleration of deep neural network training with resistive cross-point devices: design considerations. Front. Neurosci. 10:333. 10.3389/fnins.2016.00333 27493624 PMC4954855

[B7] Hu M. Li H. Wu Q. Rose G. S. Chen Y. (2012). Memristor crossbar based hardware realization of BSB recall function, in International Joint Conference on Neural Networks (Brisbane, QLD: IEEE), 1–7. 10.1109/IJCNN.2012.6252563

[B8] Ielmini D. (2018). Brain-inspired computing with resistive switching memory (RRAM): devices, synapses and neural networks. Microelectr. Eng. 190, 44–53. 10.1016/j.mee.2018.01.009

[B9] Jo S. H. Chang T. Ebong I. Bhadviya B. B. Mazumder P. Lu W. (2010). Nanoscale memristor device as synapse in neuromorphic systems. Nano Lett. 10, 1297–1301. 10.1021/nl904092h 20192230

[B10] Kenyon A. J. Munde M. S. Ng W. H. Buckwell M. Joksas D. Mehonic A. (2019). The interplay between structure and function in redox-based resistance switching. Faraday Discuss. 213, 151–163. 10.1039/C8FD00118A 30371722

[B11] Kuzum D. Jeyasingh R. G. D. Lee B. Wong H. P. (2011). Nanoelectronic programmable synapses based on phase change materials for brain-inspired computing. Nano Lett. 12, 2179–2186. 10.1021/nl201040y 21668029

[B12] LeCun Y. Cortes C. Burges C. J. (2010). The MNIST Database of Handwritten Digits. Available online at: http://yann.lecun.com/exdb/mnist/

[B13] McKee S. (2004). Reflections on the memory wall, in Proceedings of the 1st Conference on Computing Frontiers (Ischia: ACM), 162 10.1145/977091.977115

[B14] Mead C. (1989). Analog VLSI and Neural Systems. Boston, MA: Addison-Wesley Longman Publishing Co., Inc.

[B15] Mead C. (1990). Neuromorphic electronic systems. Proc. IEEE 78, 1629–1636. 10.1109/5.58356

[B16] Mehonic A. Buckwell M. Montesi L. Garnett L. Hudziak S. Fearn S. (2015). Structural changes and conductance thresholds in metal-free intrinsic SiO_*x*_ resistive random access memory. J. Appl. Phys. 117:124505 10.1063/1.4916259

[B17] Mehonic A. Cueff S. Wojdak M. Hudziak S. Jambois O. Labb C. (2012). Resistive switching in silicon suboxide films. J. Appl. Phys. 111:74507 10.1063/1.3701581

[B18] Mehonic A. Kenyon A. J. (2016). Emulating the electrical activity of the neuron using a silicon oxide RRAM cell. Front. Neurosci. 10:57. 10.3389/fnins.2016.00057 26941598 PMC4763078

[B19] Mehonic A. Munde M. Ng W. Buckwell M. Montesi L. Bosman M. (2017). Intrinsic resistance switching in amorphous silicon oxide for high performance SiO_*x*_ ReRAM devices. Microelectr. Eng. 178, 98–103. 10.1016/j.mee.2017.04.033

[B20] Mehonic A. Shluger A. L. Gao D. Valov I. Miranda E. Ielmini D.. (2018). Silicon oxide (SiO_*x*_): a promising material for resistance switching? Adv. Mater. 30:1801187. 10.1002/adma.201801187 29957849

[B21] Muller L. K. Indiveri G. (2015). Rounding methods for neural networks with low resolution synaptic weights. arXiv:1504.05767.

[B22] Munde M. S. Mehonic A. Ng W. H. Buckwell M. Montesi L. Bosman M.. (2017). Intrinsic resistance switching in amorphous silicon suboxides: the role of columnar microstructure. Sci. Rep. 7:9274. 10.1038/s41598-017-09565-8 28839255 PMC5571160

[B23] Nandakumar S. R. Le Gallo M. Boybat I. Rajendran B. Sebastian A. Eleftheriou E. (2018). A phase-change memory model for neuromorphic computing. J. Appl. Phys. 124:152135 10.1063/1.5042408

[B24] Pi S. Li C. Jiang H. Xia W. Xin H. Yang J. J.. (2019). Memristor crossbar arrays with 6-nm half-pitch and 2-nm critical dimension. Nat. Nanotechnol. 14:35. 10.1038/s41565-018-0302-0 30420759

[B25] Pickett M. D. Medeiros-Ribeiro G. Williams R. S. (2013). A scalable neuristor built with Mott memristors. Nat. Mater. 12, 114–117. 10.1038/nmat3510 23241533

[B26] Poon C. S. Zhou K. (2011). Neuromorphic silicon neurons and large-scale neural networks: challenges and opportunities. Front. Neurosci. 5:108. 10.3389/fnins.2011.00108 21991244 PMC3181466

[B27] Prezioso M. Merrikh-Bayat F. Hoskins B. D. Adam G. C. Likharev K. K. Strukov D. B. (2015). Training and operation of an integrated neuromorphic network based on metal-oxide memristors. Nature 521, 61–64. 10.1038/nature14441 25951284

[B28] Sebastian A. Le Gallo M. Burr G. W. Kim S. BrightSky M. Eleftheriou E. (2018). Tutorial: brain-inspired computing using phase-change memory devices. J. Appl. Phys. 124:111101 10.1063/1.5042413

[B29] Serb A. Bill J. Khiat A. Berdan R. Legenstein R. Prodromakis T. (2016). Unsupervised learning in probabilistic neural networks with multi-state metal-oxide memristive synapses. Nat. Commun. 7:12611. 10.1038/ncomms12611 27681181 PMC5056401

[B30] Serrano-Gotarredona T. Masquelier T. Prodromakis T. Indiveri G. Linares-Barranco B. (2013). STDP and STDP variations with memristors for spiking neuromorphic learning systems. Front. Neurosci. 7:2. 10.3389/fnins.2013.00002 23423540 PMC3575074

[B31] Stathopoulos S. Khiat A. Trapatseli M. Cortese S. Serb A. Valov I.. (2017). Multibit memory operation of metal-oxide bi-layer memristors. Sci. Rep. 7:17532. 10.1038/s41598-017-17785-1 29235524 PMC5727485

[B32] Stoliar P. Tranchant J. Corraze B. Janod E. Besland M. P. Tesler F. (2017). A leaky-integrate-and-fire neuron analog realized with a Mott insulator. Adv. Funct. Mater. 27:1604740 10.1002/adfm.201604740

[B33] Sung C. Lim S. Kim H. Kim T. Moon K. Song J.. (2018). Effect of conductance linearity and multi-level cell characteristics of TaO_*x*_-based synapse device on pattern recognition accuracy of neuromorphic system. Nanotechnology 29:115203. 10.1088/1361-6528/aaa733 29328054

[B34] Tarkov M. S. (2015). Mapping neural network computations onto memristor crossbar, in International Siberian Conference on Control and Communications (Omsk: IEEE), 1 10.1109/SIBCON.2015.7147235

[B35] Torrezan A. C. Strachan J. P. Medeiros-Ribeiro G. Williams R. S. (2011). Sub-nanosecond switching of a tantalum oxide memristor. Nanotechnology 22:485203. 10.1088/0957-4484/22/48/485203 22071289

[B36] Vose D. (2008). Risk Analysis: A Quantitative Guide. John Wiley & Sons.

[B37] Wang I. T. Chang C. C. Chiu L. W. Chou T. Hou T. H. (2016). 3D Ta/TaO_*x*_/TiO_2_/Ti synaptic array and linearity tuning of weight update for hardware neural network applications. Nanotechnology 27:365204 10.1088/0957-4484/27/36/365204 27483492

[B38] Wright C. D. Hosseini P. Diosdado J. A. V. (2013). Beyond von-Neumann computing with nanoscale phase-change memory devices. Adv. Funct. Mater. 23, 2248–2254. 10.1002/adfm.201202383

[B39] Yu S. (2018). Neuro-inspired computing with emerging nonvolatile memory. Proc. IEEE 106, 260–285. 10.1109/JPROC.2018.2790840

[B40] Yu S. Gao B. Fang Z. Yu H. Kang J. Wong H. S. P. (2013a). A low energy oxide-based electronic synaptic device for neuromorphic visual systems with tolerance to device variation. Adv. Mater. 25:12. 10.1002/adma.201203680 23355110

[B41] Yu S. Gao B. Fang Z. Yu H. Kang J. Wong H. S. P. (2013b). Stochastic learning in oxide binary synaptic device for neuromorphic computing. Front. Neurosci. 7:186. 10.3389/fnins.2013.00186 24198752 PMC3813892

