# Supplementary material for: Simulation of Inference Accuracy Using Realistic RRAM Devices
Source: Front Neurosci. 2019 Jun 12;13:593. doi: 10.3389/fnins.2019.00593 (PMC6582938; doi:10.3389/fnins.2019.00593)
Supplement: Supplementary file 1 [file Data_Sheet_1.pdf]

Supplementary Material

1 SUPPLEMENTARY DATA

1.1 Abrupt Set Process

Figure 2A shows that the set process is abrupt when using I/V sweeps. A similar effect is observed when using voltage pulses: transition from the HRS to the LRS occurs after the application of just a few voltage pulses, as demonstrated in Figure S2.

1.2 Effect of Starting Accuracy

Figure S3 explores the effect of the starting accuracy on the sensitivity to pruning. Here we define the starting accuracy as the accuracy of a neural network with discretised weights, none of which are pruned. Figure S3 analyses networks with identical architectures - two hidden layers, each containing a 100 neurons - it is just that one set of those networks was trained for fewer epochs, thus achieving lower accuracies both with continuous weights, and with discrete weights (the latter shown in the figure). It can be seen that even a difference of $\sim$1.1% in the starting accuracy can greatly affect the pruning curves. We observe that the higher the starting accuracy, the more robust the networks become to pruning.

This effect is important to take into account when analysing characteristics of ANNs, such as the number of hidden layers. When investigating different architectures, we would ideally want to have the same starting accuracy for all of them, so that it would not affect the results. This, however, is impossible to do because it is difficult to stop the training at the right moment – increases in accuracy after some epochs are often sudden and large in magnitude. As a result, we have to deal with at least small differences in starting accuracy in the simulations investigating the effect of the number of hidden layers on sensitivity to pruning. In Figure 4A, for example, the starting accuracy of a network with one hidden layer is $\sim$95.8%, while that of a network with two hidden layers is $\sim$96.8%. Regardless of the effect of lower starting accuracy, the network with one hidden layer eventually reaches a higher accuracy than the network with two hidden layers, as we prune a larger proportion of synapses. This shows that the sensitivity to pruning is dependent not only on the starting accuracy, but also on the number of hidden layers. This conclusion could not have been made, had the starting accuracy of a network with one hidden layer been higher than that of a network with two hidden layers.

1.3 Figures

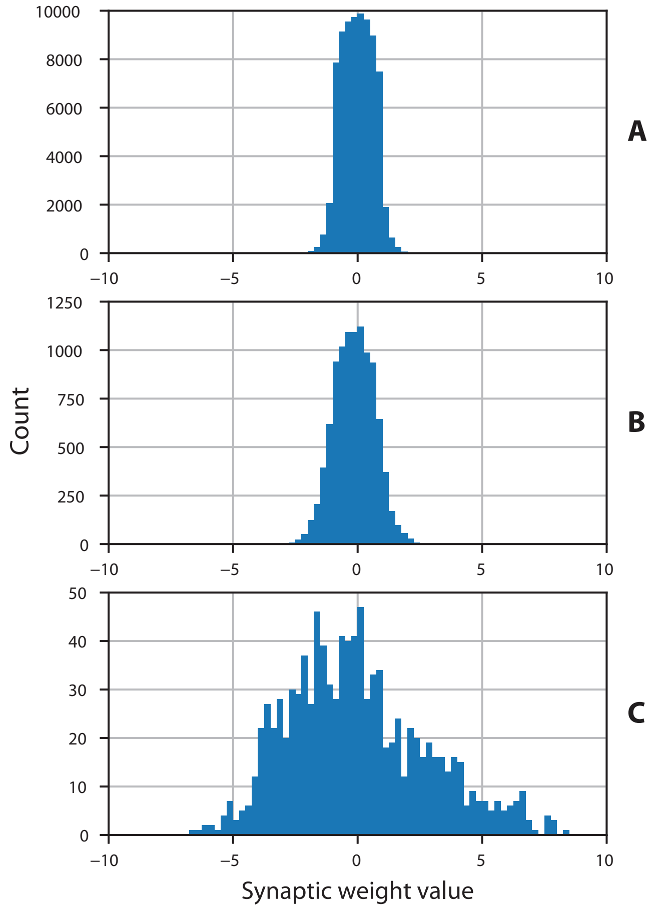

Figure S1. Typical distribution of weights in an ANN with two hidden layers. **(A)** Typical distribution of weights between the input layer and the first hidden layer. **(B)** Typical distribution of weights between the first hidden layer and the second hidden layer. **(C)** Typical distribution of weights between the second hidden layer and the output layer.

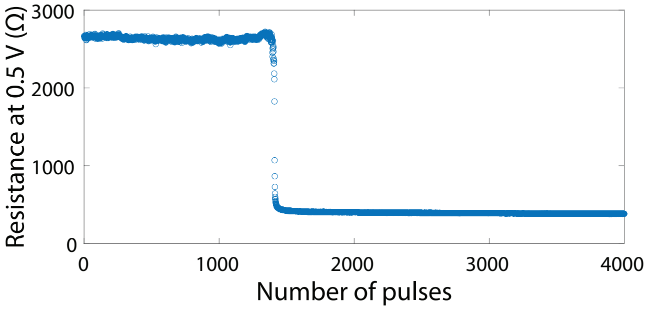

Figure S2. Potentiating characteristics obtained from the SiO_x RRAM device using voltage pulses (−1.72 V, 150 ns).

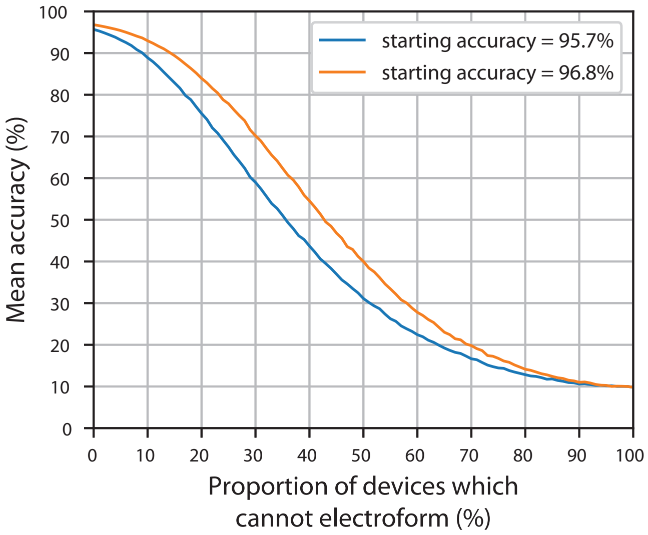

Figure S3. The effect of the starting accuracy on an ANN's sensitivity to the pruning of its synapses. Networks with 2 hidden layers each containing a 100 neurons were used; they were discretised using 10 equally spaced conductance states.

Algorithm 1 Choosing maximum and minimum discrete weights

Input: Array containing all the continuous weights in a given synaptic layer: $\mathbf{W}$, HRS/LRS ratio: $\mathrm{HRS/LRS}$, proportion of excluded synaptic weights with the largest absolute values: p_{L}

Output: Maximum discrete weight in a given synaptic layer: $w_{\text{max (discrete)}}$, minimum discrete weight in a given synaptic layer: $w_{\text{min (discrete)}}$;

procedure FIND_MAX_AND_MIN_DISCRETE_WEIGHTS($\mathbf{W}, \mathrm{HRS/LRS}, p_{\mathrm{L}}$)
    $\mathbf{W} = \mathrm{abs}(\mathbf{W})$ ▷ take absolute value of each element in the array
    $\mathbf{W} = \mathrm{desc\_sort}(\mathbf{W})$ ▷ sort array in descending order
    $s = \mathrm{size}(\mathbf{W})$ ▷ compute the size of the array
    $\mathrm{index} = \mathrm{int}(p_{\mathrm{L}} * s)$ ▷ compute the index of the maximum discrete weight
    $w_{\text{max (discrete)}} = \mathbf{W}[\mathrm{index}]$ ▷ compute maximum discrete weight
    $w_{\text{min (discrete)}} = w_{\text{max (discrete)}}/(\mathrm{HRS/LRS})$ ▷ compute minimum discrete weight
